# Horizontal positioning for accurate lateral helical blade insertion in proximal femoral nail antirotation (PFNA) for elderly osteoporotic patients

**DOI:** 10.3389/fmed.2026.1702771

**Published:** 2026-02-17

**Authors:** Yanqing Wang, Sen Dong, Hongliang Yan, Bin Ma

**Affiliations:** Department of Orthopedic Surgery, Tianjin Union Medical Center, The First Affiliated Hospital of Nankai University, Tianjin, China

**Keywords:** anteversion angle, elderly osteoporotic patients, intertrochanteric fracture, proximal femoral nail, radiation exposure

## Abstract

**Objective:**

This study aimed to investigate the horizontal positioning (HP) technique for the precise lateral insertion of the helical blade guide pin during proximal femoral nail antirotation (PFNA) procedures for elderly osteoporotic patients.

**Methods:**

This retrospective study involved elderly osteoporotic patients with femoral intertrochanteric fractures treated at Tianjin Union Medical Center from January to December 2024. Patients were categorized into the HP group and the traditional true lateral view (TLV) group. The analyzed variables included fracture classification, horizontal anteversion angle (HAA), success rate of one-time lateral pin placement (SR-OLPP), pin insertion time, frequency of intraoperative lateral fluoroscopy images (FILFI), Parker's ratio index, lag screw placement, tip-apex distance (TAD), and reduction quality.

**Results:**

A total of 86 patients participated in the study (HP group: *n* = 46; TLV group: *n* = 40), with no significant differences in baseline characteristics between the groups. The HP group demonstrated a significantly shorter pin insertion time compared to the TLV group [1 (1–3) vs. 3 (2–5) min, *p* < 0.001] and required fewer intraoperative lateral fluoroscopy images [1 (1–3) vs. 3 (2–6), *p* < 0.001]. The horizontal anteversion angle in the HP group was 6.4 ± 6.3°. The success rate of one-time lateral pin placement was higher in the HP group (95.7 vs. 85.0%, *p* = 0.138). No significant differences were found between two groups concerning reduction quality or spiral blade position and short-term post-operative complications.

**Conclusion:**

HP technology may enhance surgical efficiency and reduce radiation exposure while maintaining surgical safety and the accuracy of nail placement.

## Introduction

Femoral intertrochanteric fractures are among the most common osteoporotic injuries in the elderly, posing a substantial threat to mobility and survival (1). With the global rise in life expectancy, the incidence of these fractures continues to increase, creating significant socio-economic and healthcare burdens. Early surgical intervention is considered the standard of care, as it facilitates early mobilization and reduces complications related to prolonged immobilization (2).

Proximal femoral nail antirotation (PFNA) is now widely preferred for the treatment of intertrochanteric fractures due to its minimally invasive approach, less intraoperative blood loss, faster post-operative recovery, and favorable biomechanical stability (3). However, PFNA requires intraoperative fluoroscopic guidance to ensure accurate implant positioning—especially during insertion of the helical blade into the femoral neck (4). This dependence on fluoroscopy inevitably exposes surgeons and patients to ionizing radiation, raising occupational health concerns (5, 6). Although the risk of radiation exposure is well recognized, traditional PFNA techniques often require multiple fluoroscopic images for accurate guide pin placement, increasing cumulative radiation dose. For example, the conventional true lateral view (TLV) method may require several fluoroscopic adjustments per case, with reported median fluoroscopy exposures ranging from three to six times per procedure and associated increases in operative duration and radiation dose (7). Despite the use of protective measures, orthopedic surgeons have been shown to experience higher rates of radiation-related complications compared to the general population (8–16).

Accurate placement of the helical blade is crucial for stable fixation and prevention of complications such as implant failure or cut-out (17). On anteroposterior (AP) radiographs, the blade should be positioned in the lower third of the femoral neck; on lateral (LAT) views, it should be centrally located. Existing techniques for lateral positioning of the PFNA spiral blade—including the experience-based trial method, the TLV method, and the internal rotation method—are limited by increased operative time, repeated fluoroscopy, and variable accuracy. For instance, the TLV method is susceptible to guide pin misplacement and often requires multiple attempts, resulting in increased radiation exposure and a reported failure rate for first-attempt accurate pin placement of up to 15% (7). Advanced computer-assisted navigation systems can improve accuracy and reduce radiation, but these require substantial financial investment and specialized equipment, limiting their routine clinical application (18, 19).

To address these challenges, we introduced a novel horizontal positioning (HP) technique that utilizes intraoperative measurement of the femoral neck's horizontal anteversion angle (HAA) using standard fluoroscopy and a simple protractor. This method aims to enable precise lateral guide pin placement with minimal fluoroscopy, offering an accessible and cost-effective alternative to navigation systems.

The objective of this study is to evaluate the HP technique for the precise lateral insertion of the helical blade guide pin during PFNA procedures compared to the conventional TLV method.

## Materials and methods

### Study design and patients

This retrospective study included patients with intertrochanteric femoral fractures who underwent PFNA fixation at Tianjin Union Medical Center between January 2024 and December 2024. The inclusion criteria were: (1) patients with unilateral, closed intertrochanteric fractures and over the age of 60; and (2) those treated with PFNA. Exclusion criteria comprised: (1) pathological fractures (such as bone metastasis, primary bone tumors, or metabolic bone disease); (2) prior fractures of the affected hip; (3) bilateral femoral intertrochanteric fractures; (4) moderate to severe arthritis or femoral head necrosis on the affected side; and (5) complex intertrochanteric fractures that required open reduction due to failure of closed reduction. The study protocol was approved by the Ethics Committee of Tianjin Union Medical Center. This article is a retrospective study. Therefore, the Ethics Committee of Tianjin Union Medical Center waived the requirement to obtain distinct written informed consent from the patients.

From August 2024 onward, a novel HP technique for guide pin insertion was implemented in all eligible patients. The remaining patients, treated prior to August 2024, received the conventional TLV method, thereby establishing comparative cohorts based on the treatment period. Importantly, there were no other changes in surgical protocols, patient management, or personnel during the study period. All procedures were performed by the same experienced surgical team, and the introduction of the HP technique was the only intentional modification.

### Surgical technique

The surgical procedures were carried out by three experienced senior orthopedic trauma surgeons. Patients received either general or spinal anesthesia and were positioned on a traction table. Fractures were reduced using standard traction and rotation, confirmed by real-time X-ray (fluoroscopy), ensuring clear views while minimizing radiation. Following routine preparation, an incision was made to facilitate the insertion and proper alignment of the PFNA nail. The main difference between the patient groups was the specific method used for inserting the lateral guide pin.

*TLV Group:* after confirming correct intramedullary nail placement in the AP view, the C-arm was rotated approximately 20° from the coronal plane to align the femoral neck and shaft axes. Fluoroscopy was performed, and the guide device was adjusted until coaxial with the femoral neck. The guide pin was then inserted through a guide sleeve to ensure accurate placement.

**Figure 1:**
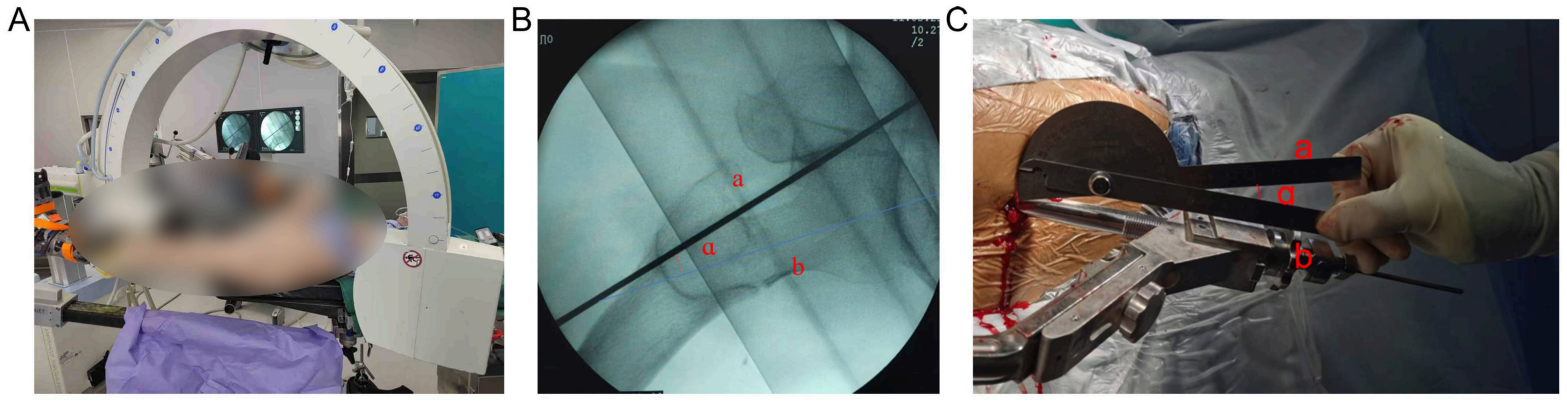
**(A)** Rotate the C-arm to achieve a true lateral view of the femoral neck. This position is confirmed when the guide wire, as visualized on the X-ray receiver, is parallel to the ground. **(B)** On the lateral view of the femoral neck, the following parameters are defined: Line a: the positioning Kirschner wire, which serves as the horizontal reference line; Line b: the central axis of the femoral neck; Angle ɑ: the angle formed between Line a and Line b, referred to as the horizontal anteversion angle. This angle represents the relationship between the central axis of the femoral neck and the horizontal plane. **(C)** The horizontal anteversion angle (ɑ) is measured using an angle measuring ruler. The horizontal arm of the ruler is aligned parallel to Line a, and the movable arm is aligned with Line b. The angle displayed between the two arms represents the horizontal anteversion angle.

*HP Group*: the C-arm was positioned parallel to the ground, and a Kirschner wire was placed horizontally at the radiation-receiving end as a reference marker (Figure 1A). Fluoroscopy was used to display both the femoral neck and the wire. The angle between the femoral neck axis and the Kirschner wire (the HAA) was measured using a protractor (GREENER Company, China; Figure 1B). After satisfactory AP positioning of the intramedullary nail, the guide device was adjusted so that the angle between the guide pin and horizontal plane matched the measured HAA (Figure 1C). The guide pin was then drilled into position, and fluoroscopy was used to confirm accurate placement in the femoral neck. If the pin was not centrally located, further adjustments were made. This technique aimed to achieve precise guide pin placement with minimal fluoroscopy, thereby reducing radiation exposure.

### Data collection

Demographic and clinical data collected included age, sex, fracture side, AO Foundation and Orthopedic Trauma Association (AO/OTA) classification, horizontal anteversion angle (HAA), success rate of one-time lateral pin placement (SR-OLPP), pin insertion time, frequency of intraoperative lateral fluoroscopy images (FILFI), post-operative radiographic outcomes [Parker's ratio, lag screw placement, and tip-apex distance (TAD)] and post-operative short-term complications. Comparative analyses were conducted between the HP and TLV groups.

Fractures were classified pre-operatively using the AO/OTA system (types 31.A1, 31.A2, and 31.A3). Intraoperative fluoroscopic images were used to assess TAD, Parker's ratio index (AP and lateral), and reduction quality. Other recorded metrics included pin insertion time, SR-OLPP, FILFI, and HAA. All radiographic measurements were corrected for magnification using the known diameter of the PFNA helical blade. The TAD was defined as the sum of distances from the tip of the helical blade to the apex of the femoral head on both AP and lateral radiographs, as described by Baumgaertner et al. (20, 21). The position of the helical blade within the femoral head was evaluated using AP and lateral Parker's ratios, with values ranging from zero to 100; lower values indicate a more inferior or posterior position (22). Reduction quality was assessed according to modified Baumgaertner criteria (20, 21); reduction was classified as good (both displacement < 4 mm and angulation < 20°, with neck-shaft angle 130–150°), acceptable (one criterion met), or poor (neither met).

### Definition of key outcome measures

The following key intraoperative outcome measures were specifically defined and recorded:

HAA: the angle between the central axis of the femoral neck and the horizontal plane (as defined by a reference Kirschner wire) on the lateral fluoroscopic view after satisfactory fracture reduction.

SR-OLPP: the percentage of cases in which the guide pin was successfully placed in the center of the femoral neck on the first lateral fluoroscopy attempt after satisfactory AP positioning. An attempt was counted only when the surgeon intentionally advanced the guide pin and subsequently obtained a lateral fluoroscopic image to verify its position.

FILFI: the total number of lateral fluoroscopic images acquired specifically for the purpose of verifying or adjusting the lateral position of the helical blade guide pin. This count explicitly excluded all fluoroscopy used for fracture reduction, assessment of nail entry point, or any other procedural step not directly related to lateral guide pin positioning. Each intentional acquisition of a lateral view to assess guide pin position was counted as one attempt.

Pin Insertion Time: defined strictly as the time interval from the confirmation of satisfactory AP guide pin position (in the middle to lower third of the femoral neck on the AP view) to the confirmation of satisfactory central position of the guide pin in the lateral view. This period encompassed only the phase of guide pin adjustment in the lateral plane and specifically excluded time for fracture reduction, nail insertion, proximal and distal locking, or any other procedural step. Timing was recorded using a standardized surgical timer.

These definitions were rigorously applied across both the HP and TLV groups by the operating surgeons and an independent data collector.

Postoperative short-term complications (within 30 days) were also analyzed, including wound infection, deep vein thrombosis, implant cut-out, intraoperative blood loss, total blood transfusion, respiratory complications (including pneumonia and pulmonary embolism), cardiac complications (including heart failure, arrhythmia, and acute coronary syndrome), and death.

### Statistical analysis

To assess the reliability of the key measurement in the HP technique, both intra-observer and inter-observer reliability analyses were conducted for the HAA. For all 46 patients in the HP group, two independent and experienced orthopedic surgeons (Observer A and Observer B), each blinded to the other's assessments, independently measured the HAA on intraoperative lateral fluoroscopy images. For intra-observer reliability, Observer A repeated the HAA measurements on the same set of images after a minimum interval of four weeks to minimize recall bias. The consistency of HAA measurements was evaluated using the intraclass correlation coefficient (ICC), calculated with a two-way mixed-effects model for absolute agreement. ICC values were interpreted as follows: less than 0.50 = poor, 0.50–0.75 = moderate, 0.75–0.90 = good, and greater than 0.90 = excellent reliability.

All statistical analyses were performed using SPSS software, version 20.0 (IBM, Armonk, NY, USA). Categorical variables were expressed as *n* (%), and continuous variables as mean ± standard deviation or median (interquartile range), as appropriate. Categorical variables were compared using the chi-square test or Fisher's exact test, while continuous variables were analyzed using the independent *t*-test or Mann–Whitney *U* test. A two-tailed *p*-value < 0.05 was considered statistically significant.

## Results

This study included a total of 86 patients with intertrochanteric fractures, comprising 46 patients in the HP group and 40 in the TLV group. The results showed no significant differences between the groups regarding age (HP: 77.5 ± 9.8 years; TLV: 77.7 ± 9.9 years; *p* = 0.964), gender distribution (Male/Female: 10/36 vs. 14/26; *p* = 0.171), fracture side (Left/Right: 29/17 vs. 22/18; *p* = 0.449), or AO/OTA fracture classification (*p* = 0.476; Table 1).

**Table 1:** Demographics data of the patients.

**Variable**	**HP (*n* = 46)**	**TLV (*n* = 40)**	***p*-value**
Age, years	77.5 ± 9.8	77.7 ± 9.9	0.964
Gender (Male/Female), *n*	10/36	14/26	0.171
Fracture side (left/right), *n*	29/17	22/18	0.449
**AO/OTA classification**, ***n*** **(%)**			0.476
31.A1	13 (28.3)	14 (35.0)	
31.A2	31 (67.4)	26 (65.0)	
31.A3	2 (4.3)	0 (0)	

AO/OTA, AO foundation and orthopedic trauma association.

The horizontal anteversion angle (HAA) in the HP group was 6.4 ± 6.3°. While the SR-OLPP was higher in the HP group (95.7%) compared to the TLV group (85.0%), this difference did not reach statistical significance (*p* = 0.138). While this suggests a potential benefit of the HP technique, the sample size may have limited the statistical power to detect a significant difference for this outcome, and thus the result should be interpreted with caution.

Notably, the HP group demonstrated significantly shorter pin insertion times compared to the TLV group [1 (1–3) vs. 3 (2–5) min, *p* < 0.001]. Similarly, the frequency of intraoperative lateral fluoroscopy images (FILFI) was significantly lower in the HP group than in the TLV group [1 (1–3) vs. 3 (2–6) times, *p* < 0.001; Table 2]. It should be noted that FILFI was used in this study as an indirect surrogate measure for intraoperative radiation exposure, as direct radiation dose measurements were not available in this retrospective analysis.

**Table 2:** Measurements of intraoperative lateral fluoroscopy.

**Variable**	**HP (*n* = 46)**	**TLV (*n* = 40)**	***p*-value**
HAA°	6.4 ± 6.3	—	
SR-OLPP (%)	44 (95.7)	34 (85.0)	0.138
Pin insertion time (min)	1 (1–3)	3 (2–5)	< 0.001
FILFI (times)	1 (1–3)	3 (2–6)	< 0.001

HAA: Horizontal anteversion angle; SR-OLPP: Success rate of one-time lateral pin placement; FILFI: Frequency of intraoperative lateral fluoroscopy images.

In the anteroposterior view, Parker's ratio index was comparable between the HP and TLV groups (39.8 ± 6.2 vs. 38.6 ± 5.6%; *p* = 0.326). Similarly, in the lateral view, the ratios were comparable (49.3 ± 5.0 vs. 49.8 ± 5.2%; *p* = 0.845). The distribution of lag screw placements in the AP view showed no significant differences, with central placement rates comparable between the HP and TLV groups (76.1 vs. 77.5%; *p* = 0.877). In the lateral view, all implants in both groups were centrally placed (100%; *p* > 0.05). Additionally, the mean TAD was comparable between the HP and TLV groups (20.4 ± 3.1 vs. 19.9 ± 2.9 mm; *p* = 0.656; Table 3).

**Table 3:** Assessment of reduction quality and the position of the spiral blade.

**Variable**	**HP (*n* = 46)**	**TLV (*n* = 40)**	***p*-value**
Parker's ratio index (AP), %	39.8 ± 6.2	38.6 ± 5.6	0.326
Parker's ratio index (LAT), %	49.3 ± 5.0	49.8 ± 5.2	0.845
**Lag screw placement (AP)**, ***n*** **(%)**			0.877
Central	35 (76.1)	31 (77.5)	
Superior	0 (0)	0 (0)	
Inferior	11 (23.9)	9 (22.5)	
**Lag screw placement (LAT)**, ***n*** **(%)**			>0.999
Anterior	0 (0)	0 (0)	
Central	46 (100)	40 (100)	
Posterior	0 (0)	0 (0)	
TAD, mm	20.4 ± 3.1	19.9 ± 2.9	0.656
**Reduction quality**, ***n*** **(%)**			0.565
Good	40 (87.0)	33 (82.5)	
Acceptable	6 (13.0)	7 (17.5)	
Poor	0 (0)	0 (0)	

AP, anteroposterior view; LAT, lateral view; TAD, tip-apex distance.

The reliability of the HAA measurement was excellent. The intra-observer reliability (test–retest) for Observer A yielded an ICC of 0.948 (95% CI: 0.907–0.971). The inter-observer reliability between Observer A (initial measurement) and Observer B was also excellent, with an ICC of 0.930 (95% CI: 0.878–0.959).

The incidence of short-term post-operative complications was significantly different between the two groups (Table 4). Specifically, no wound infections or implant cut-outs occurred in either group. The incidence of deep vein thrombosis was 17.4% in the HP group and 15.0% in the TLV group (*p* = 0.764). Intraoperative blood loss (HP group: 156.9 ± 102.3 ml, TLV group: 168.4 ± 98.9 ml; *p* = 0.597) and the proportion of patients requiring blood transfusion (HP group: 21.7%, TLV group: 22.5%; *p* = 0.932) were similar. The rates of respiratory complications (HP group: 4.3%, TLV group: 7.5%; *p* = 0.872) and cardiac complications (HP group: 6.5%, TLV group: 7.5%; *p* > 0.999) were also comparable. One patient (2.2%) in the HP group died within 30 days post-operatively, compared to none in the TLV group (*p* > 0.999).

**Table 4:** Short-term complications (within 30 days).

**Variable**	**HP (*n* = 46)**	**TLV (*n* = 40)**	***p*-value**
Wound infection	0	0	>0.999
Deep vein thrombosis	8 (17.4)	6 (15.0)	0.764
Implant cut-out	0	0	>0.999
Intraoperative blood loss (ml)	156.9 ± 102.3	168.4 ± 98.9	0.597
Total blood transfusion	10 (21.7)	9 (22.5)	0.932
Respiratory complications	2 (4.3)	3 (7.5)	0.872
Cardiac complications	3 (6.5)	3 (7.5)	>0.999
Death	1 (2.2)	0	>0.999

## Discussion

The HP technique demonstrated significant improvements in surgical efficiency, leading to a reduction in both operative time and intraoperative fluoroscopy exposure compared to the conventional method. Crucially, these advancements were achieved without compromising critical aspects of implant placement, such as tip-apex distance, or the overall quality of fracture reduction. Although the SR-OLPP was numerically higher in the HP group, this difference did not reach statistical significance. This outcome should be interpreted with caution, as the study may not have been sufficiently powered to detect a statistically significant difference given the sample size. Future studies with larger cohorts are needed to further clarify this finding.

It is important to acknowledge that the retrospective study design and the use of period-based grouping (with the HP technique introduced at a defined time point) may introduce temporal or learning-curve bias. While all surgeries were performed by the same experienced team and no changes in protocols occurred except for the introduction of HP, increased familiarity with the procedure over time could have influenced operative efficiency or outcomes. This inherent limitation should be considered when interpreting the results, and prospective randomized designs are warranted to minimize such bias in future research.

The FILFI was used as a surrogate measure to estimate and compare intraoperative radiation exposure, as the frequency of fluoroscopic images correlates directly with the radiation dose administered to both the patient and the surgical team. However, it should be emphasized that FILFI provides only an indirect estimate of radiation exposure; the actual radiation dose was not measured in this study. Thus, our findings regarding radiation reduction should be interpreted accordingly, and future studies with direct dosimetric assessment are recommended. The findings align with the growing emphasis in orthopedic trauma care on minimizing intraoperative radiation hazards. Previous studies have demonstrated that conventional methods, such as the TLV approach, often require multiple fluoroscopic adjustments for accurate guide pin placement, resulting in increased radiation exposure and operative time (7). For example, Kang et al. (5) noted that over 40% of orthopedic surgeons spend more than half their operative time using fluoroscopic guidance, with an average of 12.3 fluoroscopically guided procedures per month. The cumulative occupational risk is substantial, including increased rates of malignancy, cataracts, infertility, and other adverse effects (6, 23–25). Nelson et al. (25) reported that as few as 25 fluoroscopy images could result in patient exposures of 2,550–2,900 mR, underscoring the importance of minimizing unnecessary imaging.

A variety of strategies have been explored to reduce intraoperative radiation, including computer-assisted navigation systems, personal protective equipment, and maximizing the distance from the radiation source (18, 19). Navigation technologies, such as the ATLAS system, can improve accuracy and reduce radiation exposure but are limited by high costs, technical complexity, and the need for specialized equipment and training (18, 19). In contrast, the HP technique described in this study requires only standard operating room equipment, such as a C-arm and a simple protractor, making it a low-cost, practical, and easily implementable alternative that can be rapidly adopted in most clinical settings, especially those without access to advanced navigation systems.

The results are consistent with previous reports showing the challenges of achieving accurate guide pin placement using experience-based or TLV methods, which often require multiple fluoroscopic adjustments (7). By directly measuring the horizontal anteversion angle (HAA) after fracture reduction, the HP technique allows for individualized and precise guide pin insertion. The observed variability in HAA (6.4 ± 6.3°) highlights the importance of patient-specific measurement, as reliance on the contralateral limb or standard anatomical assumptions may be unreliable, as also demonstrated by Yu et al. (26).

Despite improved efficiency and reduced radiation exposure, the HP technique did not compromise the quality of implant placement or fracture reduction. The Parker's ratio index, tip-apex distance, and reduction quality were comparable between groups and all remained within optimal ranges, supporting the clinical safety and efficacy of the method (27, 28).

In addition to intraoperative and immediate post-operative radiographic parameters, we recorded and analyzed a range of short-term complications occurring within 30 days after surgery, including wound infection, deep vein thrombosis, implant cut-out, intraoperative blood loss, blood transfusion, respiratory and cardiac complications, and mortality (29). The incidence of these complications was low and comparable between the HP and TLV groups, indicating that the HP technique does not increase early post-operative risk. However, the study is limited by the absence of post-operative clinical and long-term follow-up outcomes, such as functional recovery, implant survival, and reoperation rates, which are essential for fully validating the sustained clinical benefits of the HP technique. We plan to address these outcomes in future prospective studies (30).

The study has several limitations. First, the HP technique may not be suitable for complex intertrochanteric fractures that require open reduction, because the HAA is a geometric relationship defined after stable fracture reduction; during open reduction and manipulation, the HAA may vary dynamically, making a single intraoperative measurement unreliable. The HP technique is therefore best suited for fractures where satisfactory closed reduction can be achieved and maintained. Second, in patients with severe osteoporosis, guide pin slippage may still occur, requiring additional adjustments and fluoroscopy. we emphasize precise surgical execution to mitigate this risk. Key steps include ensuring firm contact between the guide sleeve and the lateral cortex, using a high-speed drill with slow, controlled advancement of the guide pin, and avoiding excessive reorientation of the guide once the pin has engaged bone. These measures enhance purchase and reduce the likelihood of skiving. Third, the learning curve associated with the HP technique appears favorable. Its core principles—measuring the HAA on a true lateral fluoroscopic view and replicating this angle during guide pin insertion—are straightforward. In our experience, any surgeon familiar with PFNA and basic C-arm navigation can adopt this technique efficiently. However, we acknowledge that the proficiency and consistency demonstrated in this study were achieved by experienced trauma surgeons. Prospective studies evaluating the implementation of the HP technique among lower-volume centers or surgeons earlier in their learning curve are warranted to fully define its generalizability and training requirements. Fourth, grouping by treatment period may introduce temporal and learning-curve bias, as previously discussed. Although the HP technique was the only intentional modification and the same surgical team managed all cases, we cannot entirely exclude the possibility that increased experience over time contributed to the improved efficiency observed. This limitation should be addressed in future multicenter, randomized controlled trials. Finally, since our study was conducted at a single center by experienced trauma surgeons, the generalizability of the findings may be limited. Multi-center studies across diverse clinical settings and levels of surgeon experience are needed to validate and extend these results. As noted, the lack of long-term follow-up is another limitation. However, as the primary benefits of the HP technique are intraoperative efficiency and reduced fluoroscopy frequency (as a surrogate for radiation exposure), the absence of long-term data is less critical for evaluating this technical modification. Nevertheless, future studies should incorporate long-term and functional outcomes to provide a more comprehensive assessment. Comparative studies evaluating HP technology against other radiation reduction strategies, such as navigation systems, could further clarify its relative advantages in terms of cost-effectiveness, learning curve, and clinical outcomes.

The HP technique may reduce intraoperative fluoroscopy and surgical time without compromising clinical outcomes. Broader adoption of the HP technique may contribute to improved occupational safety in orthopedic trauma surgery, although these findings should be interpreted in light of the study's retrospective design, single-center setting, and lack of long-term outcome data.

## Data Availability

The original contributions presented in the study are included in the article/Supplementary material, further inquiries can be directed to the corresponding author.
